# Mex3a promoter hypomethylation can be utilized to diagnose HBV-associated hepatocellular carcinoma: a randomized controlled trial

**DOI:** 10.3389/fphar.2024.1325869

**Published:** 2024-11-05

**Authors:** Jie-Ru Yang, Yu-Xin Tian, Jin-E. Li, Ying Zhang, Yu-Chen Fan, Kai Wang

**Affiliations:** ^1^ Department of Hepatology, Qilu Hospital of Shandong University, Jinan, China; ^2^ Laboratory of Basic Medical Sciences, Qilu Hospital of Shandong University, Jinan, China; ^3^ Institute of Hepatology, Shandong University, Jinan, China

**Keywords:** biomarker, HBV-associated, hepatocellular carcinoma, MethyLight, Mex3a

## Abstract

**Background:**

Hepatocellular carcinoma remains a health challenge for humanity. Therefore, there is an urgent need to develop novel biomarkers with high efficiency yet fast ability to meet the requirements of hepatocellular carcinoma treatment.

**Methods:**

A total of 229 patients with HBV-associated hepatocellular carcinoma (HCC), 298 patients with chronic hepatitis B (CHB), and 96 healthy controls were retrospectively analyzed. Methylation levels of the Mex3a promoter in peripheral blood mononuclear cells (PBMCs) were measured using MethyLight to obtain clinical and laboratory parameters.

**Results:**

The Mex3a promoter methylation level in HCC patients (median: 0.289% and interquartile range: 0.126%–0.590%) was significantly lower than that in CHB patients (median: 0.999%, interquartile range: 0.417%–1.268%, and *p* < 0.001) and healthy people (median: 2.172%, interquartile range: 1.225%–3.098%, and *p* < 0.001). The Mex3a mRNA levels in HCC patients (median: 12.198 and interquartile range: 3.112–18.996) were significantly higher than those in CHB patients (median: 1.623 and interquartile range: 0.066–6.000, and *p* < 0.001) and healthy controls (median: 0.329, interquartile range: 0.031–1.547, and *p* < 0.001). MethyLight data were expressed as a percentage of the methylated reference (PMR) value. The Mex3a PMR value was negatively correlated with the mRNA expression level (Spearman’s R = −0.829 and *p* < 0.001). The Mex3a PMR value of HCC patients was significantly correlated with age (Spearman’s R = 0.113 and *p* = 0.044), and the mRNA level was significantly correlated with ALT (Spearman’s R = 0.132 and *p* = 0.046). The Mex3a promoter methylation levels and mRNA levels were also independent factors in the development of liver cancer. The Mex3a promoter methylation and mRNA levels were better at distinguishing HCC from CHB than AFP [area under the receiver operating characteristic curve (AUC) for predicting HCC vs. CHB: 0.915 vs. 0.715: *p* < 0.001]. The combined use of AFP and Mex3a methylation levels and mRNA levels further improved the area under the receiver operating characteristic curve.

**Conclusion:**

The presence of Mex3a promoter hypomethylation in hepatocellular carcinoma can be used as a non-invasive biomarker for the early detection of liver cancer.

## Introduction

Hepatocellular carcinoma (HCC) poses a global health threat as the most prevalent type of liver cancer, with its incidence increasing worldwide. It is estimated that HCC is the third most common reason of cancer-related fatalities, and viral infections are mostly to blame (Sarin et al., 2020; Lee et al., 2021). Eighty percent of those who contract the virus develop a chronic infection that eventually results in cirrhosis and HCC (Zhao et al., 2021). Despite the Chinese government implementing hepatitis B virus (HBV) vaccination programs for newborns as early as the last century, HBV-related HCC still accounts for approximately 85% of all HCC cases in China to date (Petruzziello, 2018). A previous study found that chronic HBV infection was the main cause of HCC in East Asia and sub-Saharan Africa, while hepatitis C virus (HCV) infection and alcohol abuse were significant risk factors in North America, Europe, and Japan after compiling the major risk factors from around the world. These gaps may be related to low HBV vaccine coverage in developing countries (Toh et al., 2023).

The tumor detection rate is influenced by a variety of variables. DNA methylation is crucial for controlling the expression via epigenetic regulators (Busslinger et al., 1983; Nishiyama and Nakanishi, 2021; Yisraeli et al., 1986; Zhu et al., 2022; Nagaraju et al., 2022). This interesting phenomenon has gained the attention of many researchers and medical practitioners as it has been observed to manifest itself across a plethora of malignant growths, such as gastric cancer (Ren et al., 2022), hepatocellular carcinoma (Yang et al., 2022), breast cancer (Xu et al., 2020), and esophageal squamous-cell carcinoma (Xi et al., 2022). DNA methylation in peripheral blood may provide novel biomarkers of exposure and immunity to examine cancer risk (Michaud and Kelsey, 2021). MethyLight is a sensitive real-time PCR approach that is appropriate for identifying low-frequency DNA methylation biomarkers and is very specific and sensitive compared to other techniques for measuring methylation levels (Yang et al., 2022; Kristensen et al., 2014).

The Mex-3 protein is a translational regulator that supports the preservation of germline totipotency. Mex3a to Mex3d refer to a family of four homologous human Mex3 genes (Buchet-Poyau et al., 2007). Mex3a was selected for our study because it plays an important role as a key regulator of gene expression in various cancer types, particularly in processes related to cell proliferation, differentiation, and tumor progression (Lederer et al., 2021), including lung adenocarcinoma (Liang et al., 2020), breast cancer (Wang et al., 2021), colorectal cancer (Gutierrez et al., 2021), cervical cancer (Peng et al., 2022), pancreatic ductal adenocarcinoma (Wang et al., 2020), osteosarcoma (Wang et al., 2021), glioma (Yang et al., 2021), esophageal squamous cell carcinoma (Wei et al., 2020), ovarian cancer (Wang et al., 2023), renal cell carcinoma (Qiu et al., 2022), nasopharyngeal carcinoma (Xiang et al., 2022), and liver cancer (Yang et al., 2020). However, its underlying mechanisms and regulation in HCC remain poorly understood. Previous studies have suggested that abnormal methylation of gene promoters, including Mex3a, may be associated with the onset and progression of HCC (Wang et al., 2024). However, the specific role of Mex3a promoter methylation in HCC, particularly in the context of HBV, has not been thoroughly studied.

In this study, we examined the link between the methylation state of the Mex3a promoter in patients and other clinicopathological traits using MethyLight to detect the degree of Mex3a promoter methylation in HBV-associated HCC, chronic hepatitis B (CHB), and healthy controls (HCs). The next step was to assess the potential clinical relevance of the methylation state of the Mex3a promoter as a non-invasive biomarker for the diagnosis of HCC.

## Materials and methods

### Patients and controls

The present research undertook an extensive retrospective analysis in which 96 healthy controls (HCs), 298 chronic hepatitis B (CHB) patients, and 229 hepatocellular carcinoma (HCC) patients were duly enrolled in the program at the esteemed Department of Hepatology, Qilu Hospital of Shandong University, between September 2019 and December 2021. The diagnostic criteria for HCC patients were established using the 2018 revision of the American Association for the Study of Liver Diseases (AASLD) practice guidelines for the treatment of HCC. The American Society for the Study of Liver Disorders’ 2018 amended diagnostic criteria were used to diagnose CHB and LC (AASLD).

Blood samples were excluded if the participant fit any of the categories listed below: pregnancy; coinfection with the hepatitis A, C, D, or E viruses; coexistence with other liver conditions, such as autoimmune, alcoholic, or drug-related hepatitis; metabolic issues; HIV infection; HBsAg negativity; coexistence with other malignancies; insufficient data; and withdrawal. Furthermore, sample collection was permitted (in writing) by all participants, and all research plans were approved by the Shandong University Qilu Hospital Ethics Committee (No. KYLL-202301–007) and follow the principles outlined in the 1975 Helsinki Declaration.

### Plasma collection and peripheral blood mononuclear cell isolation

On the first day after diagnosis, 5 mL of venous blood was taken from each participant, and ethylene diamine tetraacetic acid (EDTA) was used as an anticoagulant. Gradient centrifugation of PBMCs from blood was performed using Ficoll-Paque Plus (GE Healthcare, Uppsala, Sweden). The PBMCs were then recovered from the interface, washed three times with phosphate-buffered saline, and stored at −20°C until use.

### Sodium bisulfite modification

An EZ DNA Methylation-Gold Kit (Zymo Research, Orange, CA, United States) was used to execute DNA bisulfite modification. In this work, a 10-μL modified DNA solution was prepared, which can not only be directly used for MethyLight testing but can also be stored in a −20°C environment.

### TaqMan probe-based quantitative methylation-specific polymerase chain reaction (MethyLight)

The MethyLight technique was used to assess the methylation level of the Mex3a promoter. In this research, two sets of probes and primers were employed: one set for the reference gene *β-actin-1*, which is used to standardize the input DNA, and another set for the methylated Mex3a gene. Mex3a primer and probe designs for the *β-actin-1* gene were developed according to earlier publications (Yang et al., 2022). The gene sequence of the Mex3a promoter was obtained from the UCSC Genome Browser database (website: http://genome.ucsc.edu/). The number of CpG sites present in the amplified region was 1, and the length of the amplicon used to determine the percentage of the methylated reference (PMR) was 269 bp. A software program, oligo7, developed by the prestigious OLIGO 1267 Vondelpark, Colorado Springs, CO 80907, United States, was then utilized to construct the requisite reverse and forward primers, as well as the all-important probes used in this study. Table 1 lists all the sequences.

**TABLE 1:** Sequences of primers and probes used.

Gene	Forward primer sequence (5′-3′)	Reverse primer sequence (5′-3′)	Probe oligo sequence
MethyLight			
Mex3a	GGT​TTT​AAA​GGG​GTA​ATT​ATT​AAG​C	CAT​TAT​TAT​ACT​CGA​AAA​TCT​TAC​CA	ATT​ACG​GGT​GTT​TTA​GGT​AAC​GTG​GAG
*β-actin-1*	TGG​TGA​TGG​AGG​AGG​TTT​AGT​AAG​T	AAC​CAA​TAA​AAC​CTA​CTC​CTC​CCT​TAA​A	ACC​ACC​ACC​CAA​CAC​ACA​ATA​ACA​AAC​ACA
RT-qPCR			
Mex3a	TGG​AGA​ACT​AGG​ATG​TTT​CGG​G	GAG​GCA​GAG​TTG​ATC​GAG​AGC	
*β-actin-2*	ATG​GGT​CAG​AAG​GAT​TCC​TAT​GTG	CTT​CAT​GAG​GTA​GTC​AGT​CAG​GTC	

The total volume of MethyLight assays was 10 μL, containing 5 μL of MethyLight Master Mix, which consisted of HotStarTaq Plus DNA Polymerase, EpiTect Probe PCR Buffer and a dNTP mix (dATP, dCTP, dGTP, and dTTP), 2 μL of nuclease-free water, 0.4 μL of forward and reverse primers, 0.2 μL of TaqMan probe, and 2 μL of bisulfite-converted DNA. The following conditions were used to execute MethyLight using a Stratagene Mx3005P instrument (Stratagene, La Jolla, CA) from Agilent Technologies. Fifty cycles of 95°C for 15 s and 60°C for 1 min each were performed after 15 min at 95°C. As a standard for methylation, human control DNA (QIAGEN, Hilden, Germany) with modified CpG methylation was produced *in vitro* using SssI methylase and bisulfite. A PMR characterized the MethyLight data. Each sample was tested three times. Each plate had both negative and positive controls and at least three control wells without a template. PMR = 100% × 2 exp−[Delta Ct (target gene in sample-control gene in sample)-(Delta Ct 100% methylated target in reference sample-control gene in reference sample)]c (Yang et al., 2022).

### Quantitative real-time polymerase chain reaction

TRIzol (Invitrogen, Carlsbad, CA, United States) was used to extract total RNA from PBMCs. An Eppendorf BioPhotometer (Brinkmann Instruments, Westbury, NY) was employed to determine the RNA concentration, followed by the creation of cDNAs from RNA using reverse transcription and a first-strand cDNA synthesis kit. Real-time PCR Mex3a assessment of Mex3a mRNA expression was performed using the LightCycler 480 System (Roche Diagnostics, Mannheim, Germany) and SYBR Green (Toyobo, Osaka, Japan). *β-actin-2* served as the internal reference. In a total volume of 10 μL, 1 μL cDNA, 0.5 μL of each primer, 3 μL water, and 5 μL SYBR Green were used for amplification. Table 1 provides a description of the primers. Following the first step of 95°C for 30 s, the PCR reaction was carried out in 50 cycles of 95°C for 5 s, 55°C for 30 s, and a final step of 72°C for 30 s. Each sample underwent comparative real-time RT-PCR experiments in triplicate. The comparative 2 (–∆∆Ct) technique was used to calculate the Mex3a mRNA levels.

### Clinical data collection

Standard techniques were used at the laboratory of Shandong University Qilu Hospital to identify the following markers. Aspartate albumin (ALB), aminotransferase (AST), AFP, alanine aminotransferase (ALT), total bilirubin (TBIL), HBV-DNA burden, and HBeAg were among the serum biochemical indicators (COBAS Integra 800; Roche Diagnostics). Hemostasis indicators (ACL TOP 700; Instrument Laboratory, Lexington, MA, United States) included prothrombin time activity (PTA) and prothrombin time-international normalized ratio (PT-INR). All imaging results, including magnetic resonance imaging data and computed tomography, were diagnosed by a radiologist who did not know the characteristics of the patient. All tissue specimens were judged by a pathologist who did not know the characteristics of the patient, which were collected along with the patient records of histopathological data including vascular infiltration and tumor size. According to the Child–Pugh classification and the symptoms present upon hospital admission, liver function assessment was executed. Additionally, all HCC patients were divided into two subgroups based on Barcelona (BCLC) staging, including the early stages 0 and A and the last stages C and D.

### Statistical analysis

The collected data were analyzed using IBM SPSS version 26.0 (SPSS Inc., Chicago, IL, United States). The Kolmogorov–Smirnov test allows us to determine whether a sample data distribution typically matches the characteristics of a normal distribution. Median values (25th percentile; 75th percentile) and categorical variables were used to express quantitative variables and resolved as numbers (%), respectively. The Kruskal–Wallis H test and the Mann–Whitney *U* test were used to compare quantitative variables. In order to reduce selection bias in the observed data, an effective statistical technique (propensity score matching) was employed in this study. A caliper width of 0.02 was employed with a one-to-one matching strategy, ensuring that each individual in the treatment group was matched with exactly one individual in the control group based on the similarity within this specified caliper width. The resulting matched pairs were then utilized in the subsequent analysis. Two-group comparisons of the PMR for the Mex3a promoter and Mex3a mRNA levels in HCC, CHB, and HC groups were performed using the Kruskal–Wallis test. The relationship between the methylation level of Mex3a and the quantitative clinical data was investigated using the Spearman’s test. The area under the receiver operating characteristic curve (AUC) was used to evaluate the diagnostic usefulness of the Mex3a methylation level and the AFP score in the diagnosis of HCC patients, i.e., separating HCC patients from CHB patients in our case. Moreover, a model based on binary logistic regression was presented to evaluate the usefulness of combining the diagnosis of AFP and Mex3a methylation levels. The highest Youden index, or Youden index-based cutoff point, was calculated using the coordinates of the receiver operating characteristic (ROC) curve. The following indices were used to assess the diagnostic accuracy: positive predictive value (PPV), sensitivity, specificity, and negative predictive value (NPV). The analysis of several logistic regressions was utilized to pinpoint separate risk variables for liver cancer. All statistical analyses were two-sided, with a *p*-value <0.05 considered statistically significant.

## Results

### General characteristics of subjects

**FIGURE 1:**
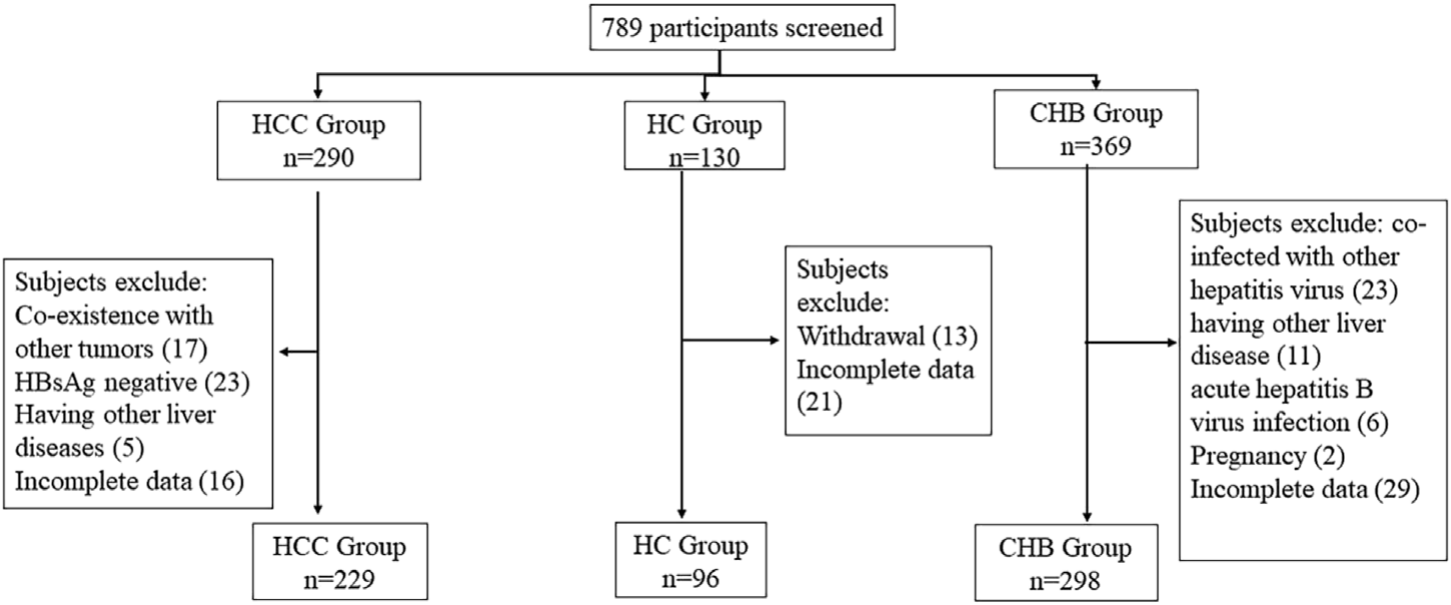
Patient selection process.

The detailed screening process of the participants is demonstrated in Figure 1. A total of 789 participants were initially screened, but eventually, 623 individuals were accepted, including 229 HCC patients, 298 CHB patients, and 96 healthy controls (HCs). The initial characteristics of the enrolled individuals are listed in Table 2.

**TABLE 2:** Baseline characteristics of the enrolled participants.

Variable	HCC group (n = 229)	CHB group (n = 296)	HC group (n = 96)
Age (years)	56 (49–63)	48 (38–55)	40 (32–55.75)
Male, n (%)	106 (79.7)	119 (72.6)	11 (42.3)
ALT (U/L)	32.00 (21.00–47.00)	41.00 (25.50–104.50)	15.00 (10.00–23.00)
AST (U/L)	36.00 (26.00–59.00)	44.00 (25.00–99.00)	21.00 (15.00–41.50)
TBIL (μmol/L)	17.25 (12.93–25.35)	17.00 (11.90–39.45)	13.90 (8.40–33.80)
ALB (g/L)	40.90 (36.40–44.15)	41.40 (33.60–46.40)	46.95 (45.70–48.88)
PLT (10 ^ 9/L)	127 (88.50–186.00)	148.5 (80.00–201.00)	238 (210.00–290.00)
PT-INR	12.7 (11.8–13.55)	12.5 (11.6–14.98)	NA
PTA (%)	86 (75–96)	87 (68–100)	NA
AFP (ng/mL)	31.40 (5.513–753.30)	6.47 (2.72–62.86)	NA
HBV-DNA (+), n (%)	122 (53.3)	214 (72.3)	NA
HBeAg (+), n (%)	218 (95.2)	291 (98.3)	NA
Encephalopathy (%)	68 (29.69)	131 (44.26)	NA
Ascites (%)	11 (4.80)	26 (8.78)	NA

Quantitative variables are expressed as the median (25th percentile; 75th percentile). Categorical variables are expressed as numbers (%). HCC, hepatocellular carcinoma; CHB, chronic hepatitis B; HC, healthy control; HBV, hepatitis B virus; AFP, alpha-fetoprotein; AST, aspartate aminotransferase; ALT, alanine aminotransferase; ALB, albumin; TBIL, total bilirubin; INR, international normalized ratio; PTA, prothrombin time activity; HBeAg, hepatitis B e surface antigen; NA, not available.

### Methylation status of the Mex3a promoter in different groups

**FIGURE 2:**
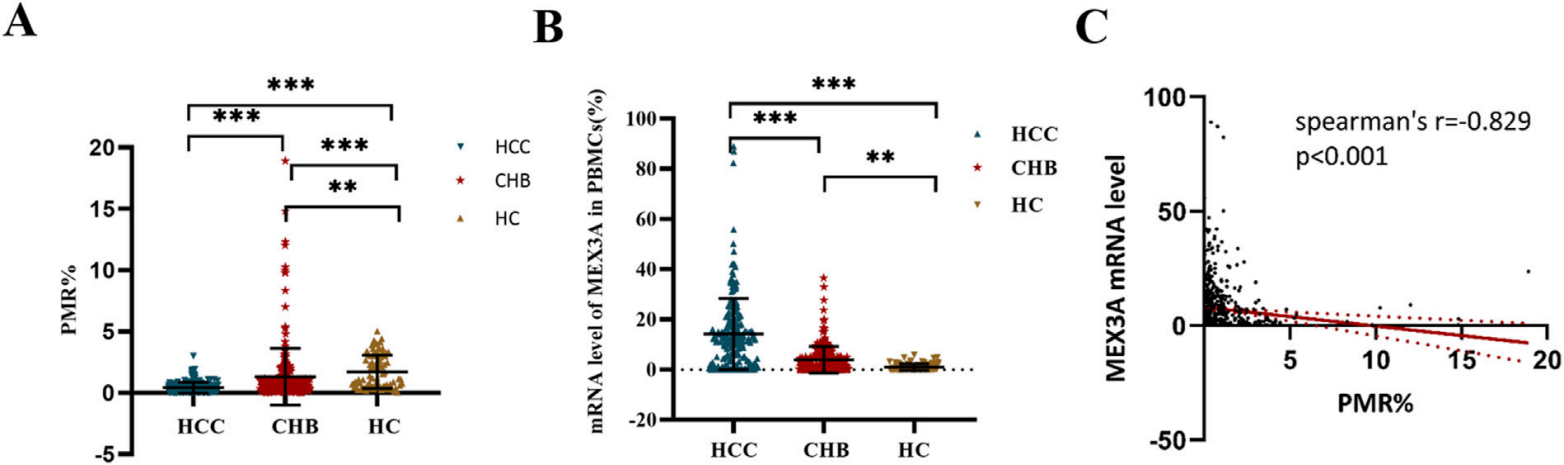
Mex3a distribution of mRNA expression and methylation levels among participants. **(A)** Mex3a promoter methylation in patients with HCC, CHB, and HCs associated with HBV; ***p < 0.001. **(B)** Mex3a mRNA levels in patients with HCC, patients with CHB, and HCs related to HBV; ***p < 0.001, **p < 0.01. The expression levels of Mex3a mRNA are normalized to ACTB, and the HC is set as the baseline, with its expression level standardized to 100%. **(C)** Relationships between the mRNA level in PBMCs and the level of Mex3a promoter methylation. Two-group comparisons of the PMR for the Mex3a promoter and Mex3a mRNA levels in HCC, CHB, and HC groups were performed using the Kruskal–Wallis test.

Figure 2A displays the PMR values used to represent the methylation status of the Mex3a promoter in various participant groups. The PMR value for the Mex3a promoter was remarkably lower in patients with HBV-associated HCC (median: 0.289% and interquartile range: 0.126%–0.590%) than in those with CHB (median: 0.999%, interquartile range: 0.417%–1.268%, and *p* < 0.001) and significantly lower than in HCs (median: 2.172%, interquartile range: 1.225%–3.098%, and *p* < 0.001), as determined by the Kruskal–Wallis test. In contrast, HCs outperformed CHB patients in terms of the Mex3a promoter’s amount of methylation (*p* < 0.001).

### Mex3a mRNA levels in different groups

The RT-PCR analysis results are displayed in Figure 2B. Mex3a mRNA expression was observed to significantly increase with disease progression. More specifically, patients with HCC (median: 12.198 and interquartile range: 3.112–18.996) showed a remarkably higher level of Mex3a mRNA than patients with CHB (median: 1.623, interquartile range 0.066–6.000, and *p* < 0.001) and HCs (median: 0.329, interquartile range 0.031–1.547, and *p* < 0.001). The Mex3a mRNA level in the patients with CHB was also higher than that in the HCs (*p* = 0.003). Subsequently, Spearman rank correlation analysis was introduced into this study to further examine the underlying association between Mex3a methylation levels and Mex3a mRNA expression levels. As shown in Figure 2C, the PMR value of Mex3a was negatively correlated with mRNA expression levels (Spearman’s R = −0.829 and *p* < 0.001).

### Associations between Mex3a promoter methylation levels and clinicopathological features in HCC

**FIGURE 3:**
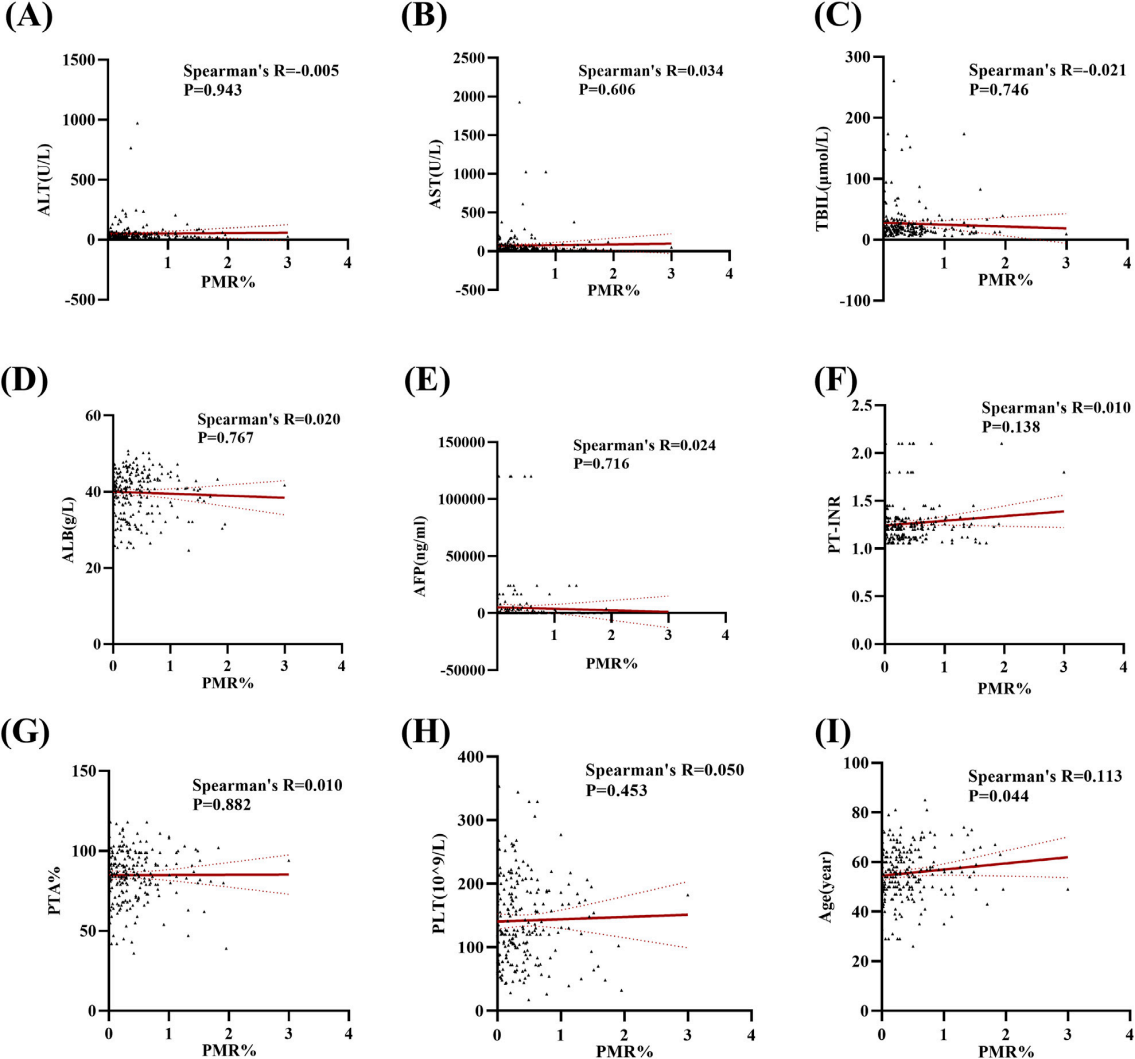
Methylation levels at the Mex3a promoter and quantitative clinical data in the HCC group are correlated. **(A)** Correlation between PMR values of the Mex3a promoter and ALT in patients with liver cancer. **(B)** Correlation between PMR values of the Mex3a promoter and AST in patients with liver cancer. **(C)** Correlation between PMR values of Mex3a promoter and TBIL in patients with liver cancer. **(D)** Correlation between PMR values of the Mex3a promoter and ALB in patients with liver cancer. **(E)** Correlation between PMR values of the Mex3a promoter and AFP in patients with liver cancer. **(F)** Correlation between PMR values of Mex3a promoter and PT-INR in patients with liver cancer. **(G)** Correlation between PMR values of the Mex3a promoter and PTA% in patients with liver cancer. **(H)** Correlation between PMR values of the Mex3a promoter and PLT in patients with liver cancer. **(I)** Correlation between PMR values of the Mex3a promoter and age in patients with liver cancer. The relationship between the methylation level of Mex3a and the quantitative clinical data was investigated using the Spearman’s test.

In this section, the relationship between Mex3a promoter methylation levels and clinical parameters in HBV-associated HCC patients was carefully analyzed. As depicted in Table 3, the Mex3a promoter methylation level was significantly higher in those aged over 55 years (median: 0.309% and interquartile range: 0.142%–0.636%) than in those aged equal to and under 55 years (median: 0.260%, interquartile range: 0.059%–0.496%, and *p* = 0.050), and the level of Mex3a promoter methylation was lower in HBV-DNA positive patients (median: 0.244% and interquartile range: 0.067%–0.491%) than in HBV-DNA negative patients (median: 0.323%, interquartile range 0.170%–0.624%, and *p* = 0.024). Meanwhile, the Mex3a promoter methylation level was significantly higher in patients with ascites (median: 0.438% and interquartile range: 0.178%–0.678%) than in those without ascites (median: 0.252%, interquartile range: 0.086%–0.502%, and *p* = 0.008). The level of Mex3a promoter methylation exhibits negligible correlation with gender (*p* = 0.667), HBeAg (*p* = 0.586), AFP (Ng/mL) (*p* = 0.907), primary tumor number (*p* = 0.817), tumor size (*p* = 0.265), lymph node metastasis (*p* = 0.858), distant metastasis (*p* = 0.420), vascular invasion (*p* = 0.328), CTP staging (*p* = 0.648), BCLC staging (*p* = 0.237), or encephalopathy (*p* = 0.079). Then, Spearman rank correlation was used to test the relationship between Mex3a promoter methylation and ALT, AST, TBIL, ALB, AFP, PT-INR, PTA%, PLT (10^9/L), and age. The Mex3a PMR values of HCC patients were correlated with age (Spearman’s R = 0.113 and *p* = 0.044). However, the Mex3a promoter methylation level was not correlated with ALT (Spearman’s R = −0.005 and *p* = 0.943), AST (Spearman’s R = 0.034 and *p* = 0.606), TBIL (Spearman’s R = −0.021 and *p* = 0.746), ALB (Spearman’s R = 0.020 and *p* = 0.767), AFP (Spearman’s R = 0.024 and *p* = 0.716), PT-INR (Spearman’s R = 0.010 and *p* = 0.138), PTA% (Spearman’s R = 0.010 and *p* = 0.882), or PLT (10^9/L) (Spearman’s R = 0.050 and *p* = 0.453) (Figure 3).

**TABLE 3:** Associations between Mex3a promoter methylation level and clinicopathological features in HCC.

Parameters	Total number	PMR (%)	*p*-value
Gender			0.667^a^
Male	147	0.296 (0.129–0.590)	
Female	82	0.289 (0.087–0.618)	
Age (year)			0.05^a^*
≤55	111	0.260 (0.059–0.496)	
>55	118	0.309 (0.142–0.636)	
HBeAg			0.586^a^
Negative	110	0.296 (0.139–0.604)	
Positive	119	0.276 (0.104–0.551)	
HBV-DNA			0.024^a^ ^**^
Negative	124	0.323 (0.170–0.624)	
Positive	105	0.244 (0.067–0.491)	
AFP (ng/mL)			0.907^a^
≤20	96	0.292 (0.115–0.514)	
>20	133	0.283 (0.126–0.618)	
Primary tumor number			0.817^a^
Single	104	0.296 (0.108–0.624)	
Multiple	125	0.276 (0.127–0.516)	
Tumor size			0.265^a^
≤ 5 cm	152	0.296 (0.132–0.590)	
>5 cm	77	0.244 (0.107–0.552)	
Lymph node metastasis			0.858^a^
Yes	24	0.367 (0.063–0.675)	
No	205	0.289 (0.131–0.589)	
Distant metastasis			0.420^a^
Yes	19	0.295 (0.129–0.604)	
No	210	0.244 (0.074–0.476)	
Vascular invasion			0.328^a^
Negative	133	0.310 (0.126–0.611)	
Positive	96	0.242 (0.115–0.487)	
CTP staging			0.648^b^
A	166	0.279 (0.104–0.561)	
B	53	0.310 (0.183–0.625)	
C	10	0.314 (0.066–0.772)	
BCLC staging			0.237^a^
0/1/2	111	0.311 (0.142–0.618)	
3/4	118	0.245 (0.107–0.505)	
Ascites			0.008^a^ ^**^
No	161	0.252 (0.086–0.502)	
Yes	68	0.438 (0.178–0.678)	
Encephalopathy			0.079^a^
No	218	0.283 (0.107–0.561)	
Yes	11	0.449 (0.246–0.647)	

CTP, Child–Turcotte–Pugh; BCLC, Barcelona clinic liver cancer.

*p < 0.05.

**p < 0.01.

***p < 0.001.

^a^ Mann–Whitney *U* test.

^b^ Kruskal–Wallis H test.

### Associations between Mex3a mRNA levels and clinicopathological features in HCC

**FIGURE 4:**
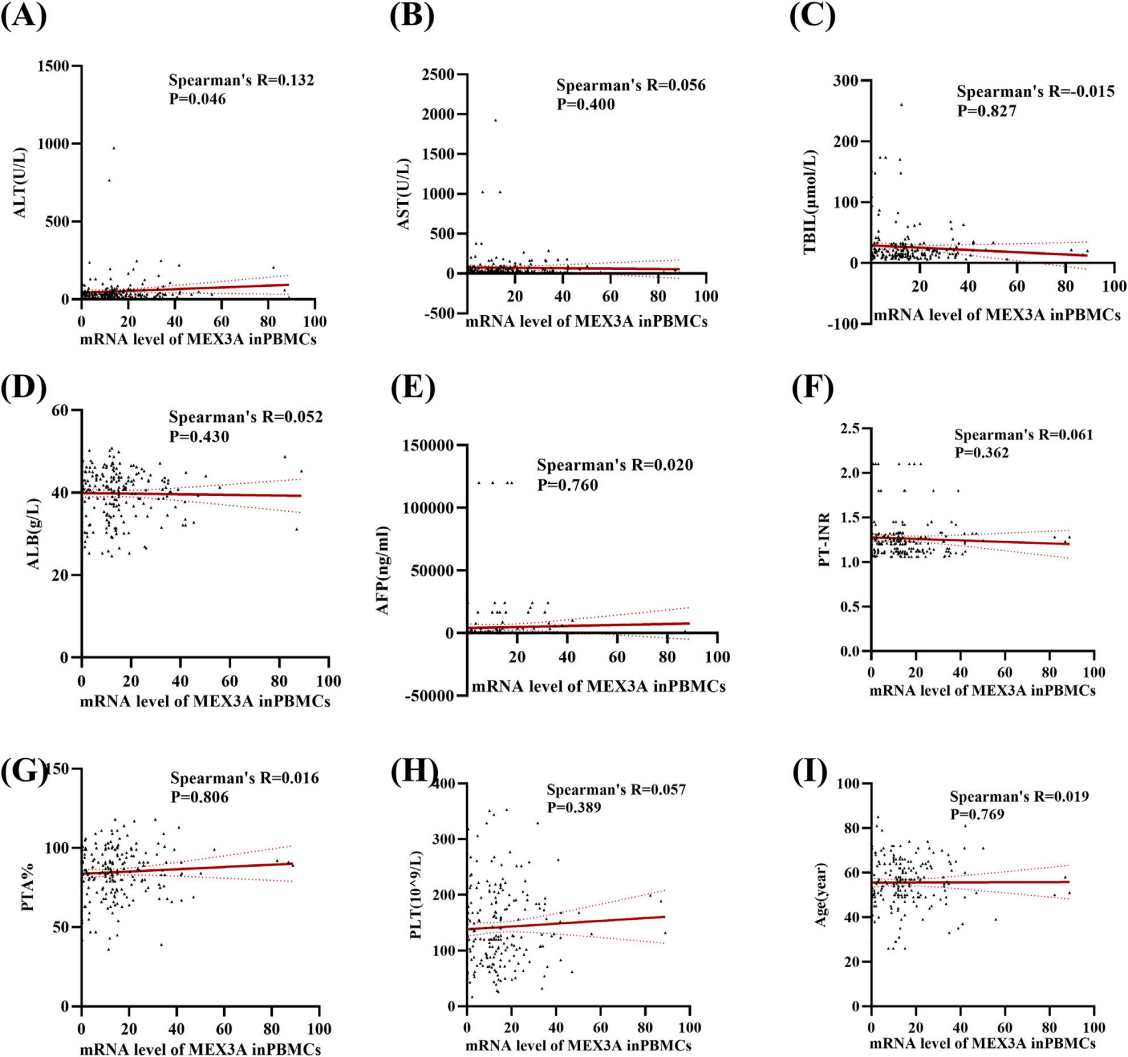
Relationships between Mex3a mRNA levels and quantitative clinical data in the HCC group. **(A)** Correlation between Mex3a mRNA levels and ALT in HCC patients. **(B)** Correlation between Mex3a mRNA levels and AST in HCC patients. **(C)** Correlation between Mex3a mRNA levels and TBIL in HCC patients. **(D)** Correlation between Mex3a mRNA levels and ALB in HCC patients. **(E)** Correlation between Mex3a mRNA levels and AFP in HCC patients. **(F)** Correlation between Mex3a mRNA levels and PT-INR in HCC patients. **(G)** Correlation between Mex3a mRNA levels and PTA% in HCC patients. **(H)** Correlation between Mex3a mRNA levels and PLT in HCC patients. **(I)** Correlation between Mex3a mRNA levels and age in HCC patients. The relationship between the Mex3a mRNA level and the quantitative clinical data was investigated using the Spearman’s test.

We then further examined correlations between Mex3a mRNA levels and clinical parameters in HBV-associated HCC patients. Table 4 demonstrates that there were no significant differences in the level of Mex3a mRNA based on gender (*p* = 0.313), HBeAg (*p* = 0.432), HBV-DNA (*p* = 0.613), AFP (Ng/mL) (*p* = 0.611), primary tumor number (*p* = 0.680), tumor size (*p* = 0.986), distant metastasis (*p* = 0.455), lymph node metastasis (*p* = 0.352), vascular invasion (*p* = 0.833), CTP staging (*p* = 0.565), BCLC staging (*p* = 0.912), ascites (*p* = 0.769), encephalopathy (*p* = 0.974), or age (*p* = 0.391). Then, Spearman rank correlation was used to test the relationship between Mex3a mRNA levels and ALT, AST, TBIL, ALB, AFP, PT-INR, PTA%, PLT (10^9/L), and age. The Mex3a mRNA level of HCC patients was correlated with ALT (Spearman’s R = 0.132 and *p* = 0.046). However, there was no correlation between the level of Mex3a promoter methylation and AST (Spearman’s R = 0.056 and *p* = 0.400), TBIL (Spearman’s R = −0.015 and *p* = 0.827), ALB (Spearman’s R = 0.052 and *p* = 0.430), AFP (Spearman’s R = 0.020 and *p* = 0.760), PT-INR (Spearman’s R = 0.061 and *p* = 0.362), PTA% (Spearman’s R = 0.016 and *p* = 0.806), PLT (10^9/L) (Spearman’s R = 0.057 and *p* = 0.389), and age (Spearman’s R = 0.019 and *p* = 0.769) (Figure 4).

**TABLE 4:** Associations between Mex3a mRNA levels and clinicopathological features in HCC.

Parameter	Total number	mRNA level (%)	*p*-value
Gender			0.313^a^
Male	147	12.692 (3.291–20.372)	
Female	82	11.134 (2.413–16.652)	
Age (year)			0.391^a^
≤55	111	11.949 (1.622–18.050)	
>55	118	12.885 (3.670–20.031)	
HBeAg			0.432^a^
Negative	110	12.253 (3.137–21.190)	
Positive	119	12.076 (3.027–16.536)	
HBV-DNA			0.613^a^
Negative	124	12.175 (3.009–18.630)	
Positive	105	12.221 (3.407–20.501)	
AFP (ng/mL)			0.611^a^
≤20	96	11.400 (1.560–16.988)	
>20	133	12.221 (3.598–20.051)	
Primary tumor number			0.680^a^
Single	104	12.309 (3.037–20.687)	
Multiple	39	12.050 (2.751–18.384)	
Tumor size			0.986^a^
≤5 cm	152	12.065 (3.195–18.956)	
>5 cm	77	12.310 (2.457–20.192)	
Lymph node metastasis			0.352^a^
Yes	24	12.991 (9.259–20.031)	
No	205	12.076 (3.009–18.930)	
Distant metastasis			0.455^a^
Yes	19	10.010 (0.253–19.009)	
No	210	12.265 (3.163–19.051)	
Vascular invasion			0.833^a^
Negative	133	12.055 (3.112–20.231)	
Positive	96	12.368 (3.061–16.988)	
CTP staging			0.565^b^
A	166	12.382 (2.522–19.533)	
B	53	12.198 (3.407–18.388)	
C	10	4.835 (0.254–19.658)	
BCLC staging			0.912^a^
0/1/2	111	12.055 (2.587–20.090)	
3/4	118	12.368 (3.137–17.439)	
Ascites			0.769^a^
No	161	12.221 (3.112–19.118)	
Yes	68	12.124 (3.061–18.976)	
Encephalopathy			0.974^a^
No	218	12.209 (3.053–18.990)	
Yes	11	10.397 (3.455–25.010)	

CTP, Child–Turcotte–Pugh; BCLC, Barcelona Clinic Liver Cancer.

*p < 0.05.

**p < 0.01.

***p < 0.001.

^a^ Mann–Whitney *U* test.

^b^ Kruskal–Wallis H test.

### Independent risk factors for HBV-associated HCC development

The risk variables for HBV-associated HCC were evaluated using multivariate logistic regression analysis. Based on the maximum Youden index, the Mex3a promoter methylation level was divided into two subgroups using 0.824% as the best cutoff point. Similarly, the Mex3a mRNA level was divided into two subgroups using 9.005% as the best cutoff point. The AFP level was divided into two subgroups using 20 ng/ml as the clinically common cutoff point. Although batch effects may introduce variability, our analysis indicates that the selected methylation cutoff point remains robust and can be used to distinguish HCC across different batches. We employed strict quality control measures and standardization techniques to mitigate batch effects, ensuring that the methylation markers retain their diagnostic value even under different experimental conditions. As shown in Table 5, all data indicated that male gender (OR = 0.342, 95% CI 0.212–0.550, and *p* < 0.001), PMR value of the Mex3a promoter>0.824% (OR = 0.189, 95% CI 0.113–0.316, and *p* < 0.001), Mex3a mRNA level <9.005% (OR = 0.035, 95% CI 0.020–0.062, and *p* < 0.001), AFP< 20 ng/mL (OR = 0.267, 95% CI 0.160–0.447, and *p* < 0.001), and HBV-DNA positivity (OR = 1.708, 95% CI 1.031–2.828, and *p* = 0.038) were independent risk variables that had an impact on the development of HCC linked to HBV.

**TABLE 5:** Independent risk factors for the development of HCC.

Variable	OR	95% CI	*p*-value
Gender (male)	0.342	0.212–0.550	<0.001
AFP (<20 ng/mL)	0.267	0.160–0.447	<0.001
Mex3a mRNA level (<9.005%)	0.035	0.020–0.062	<0.001
Mex3a PMR (>0.824%)	0.189	0.113–0.316	<0.001
HBV-DNA (+)	1.708	1.031–2.828	= 0.038

### Diagnostic value of Mex3a promoter methylation level and mRNA level

**FIGURE 5:**
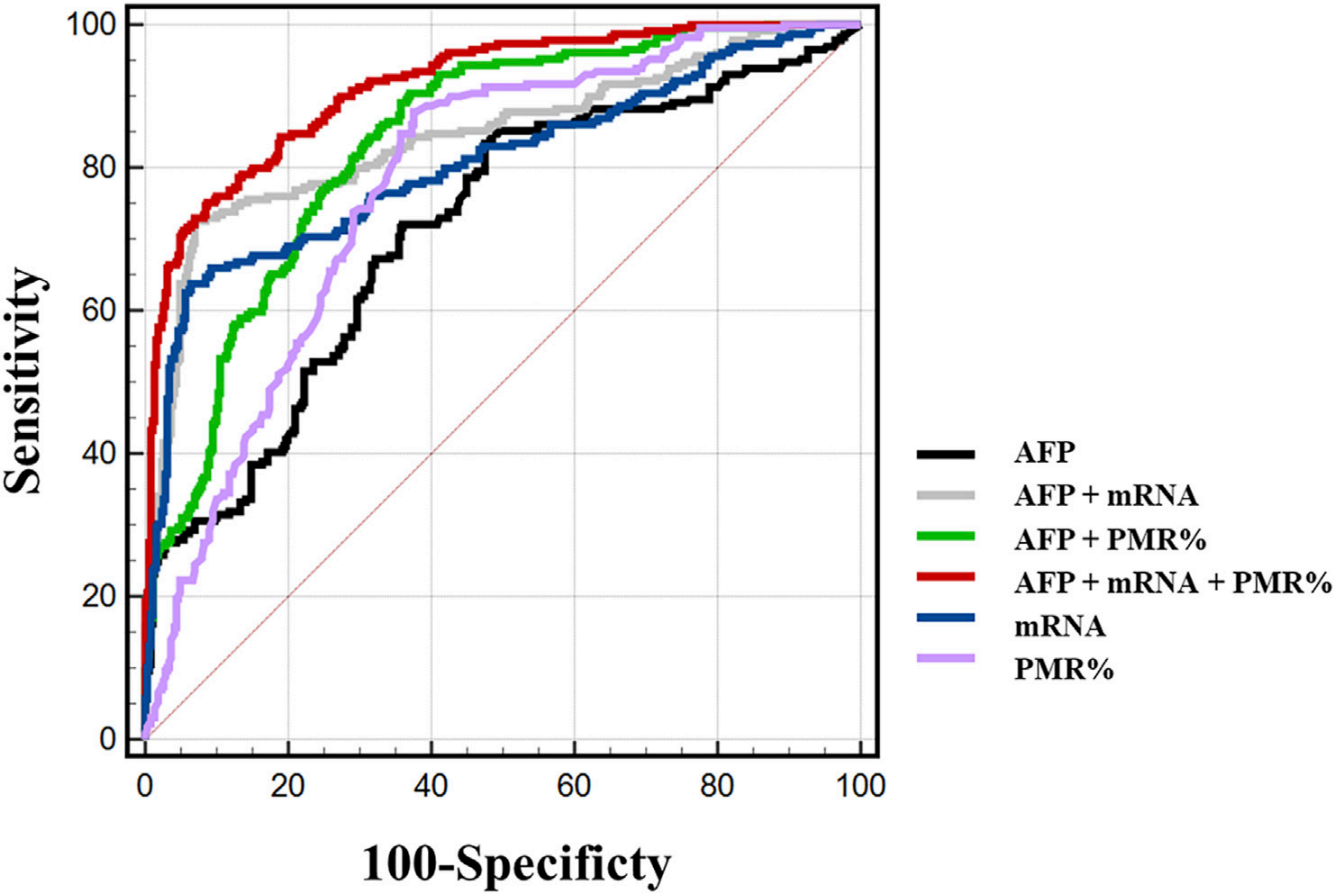
Mex3a promoter methylation and mRNA levels are useful indicators of HBV-associated HCC.

We carefully considered combinations of any two and all three indicators, Mex3a promoter methylation levels, Mex3a mRNA levels, and AFP, and used ROC curves to confirm the clinical diagnostic value of these indicators. Table 6 provides a detailed display of the experimental data. The chosen threshold was 0.354, the sensitivity was 87.8%, and the specificity was 62.5%. The AUC of the Mex3a PMR value (AUC = 0.777 and 95% CI: 0.740–0.813) was greater than that of AFP (AUC = 0.715, 95% CI: 0.672–0.757, and *p* = 0.032). In comparison to AFP (AUC = 0.715, 95% CI 0.672–0.757, and *p* = 0.002), the Mex3a mRNA level’s AUC (AUC = 0.806 and 95% CI: 0.767–0.844) was substantially higher. The sensitivity, specificity, and chosen threshold were all 72.5%, 93.6%, and 9.005%, respectively. To assess the diagnostic value of Mex3a promoter methylation levels in combination with AFP, Mex3a mRNA levels in combination with AFP, and Mex3a PMR value in combination with mRNA levels and AFP, a model based on multiple logistic regressions was created. The PMR of Mex3a and AFP combined detection AUC was 0.836 (95% CI: 0.805–0.868 and *p* < 0.0001), with a sensitivity of 90.4% and a specificity of 63.0%. The combined detection AUC of Mex3a and AFP was 0.845 (95% CI: 0.810–0.880 and *p* < 0.0001), with a sensitivity of 72.5% and a specificity of 93.1%. The combined detection AUC of PMR of Mex3a, AFP, and Mex3a mRNA level combined was 0.915 (95% CI: 0.892–0.937 and *p* < 0.0001), with a sensitivity of 75.1% and a specificity of 91.3%. The diagnostic efficiency of the combined diagnosis of the three indexes was significantly higher than that of AFP alone (*p* < 0.0001), and the specificity and sensitivity were improved significantly (Figure 5).

**TABLE 6:** Diagnostic values of Mex3a methylation level, Mex3a mRNA level, and the combined determination with AFP for distinguishing HBV-associated HCC from CHB.

Parameter	Sensitivity	Specificity	Youden index	AUC	95% CI	*p*-value compared to AFP
Mex3a mRNA level	63.8	93.6	0.574	0.806	0.767–0.844	0.0021
Mex3a PMR	87.8	62.5	0.503	0.777	0.740–0.813	0.0327
AFP	51.5	74.0	0.555	0.715	0.672–0.757	—
Mex3a mRNA level + AFP	72.5	93.1	0.656	0.845	0.810–0.880	<0.0001
Mex3a PMR + AFP	90.4	63.0	0.533	0.836	0.805–0.868	<0.0001
Mex3a mRNA level + Mex3a PMR + AFP	75.1	91.3	0.664	0.915	0.892–0.937	<0.0001

All data were sampled at six distances, and a total of 100 specimens were collected to form the verification set. The sensitivity and specificity of AFP diagnosis in the validation set were 64.1% and 72.1%, respectively, while the sensitivity and specificity of AFP diagnosis combined with the Mex3a PMR and mRNA level were 69.2% and 93.4%, respectively, which were much higher than those of AFP diagnosis alone, as shown in Table 7.

**TABLE 7:** Diagnostic values of Mex3a methylation level, Mex3a mRNA level, and the combined determination with AFP for distinguishing HBV-associated HCC from CHB in the verification set.

Parameter	Sensitivity	Specificity	Youden index
AFP	64.1	72.1	0.362
Mex3a mRNA level + Mex3a PMR + AFP	69.2	93.4	0.627

## Discussion

Characterized by nonspecific early symptoms and challenging early detection, HCC is a common cancer tumor of the digestive system known for its aggressive growth and early metastasis (Zou et al., 2022; Kim and Viatour, 2020). Due to limitations in research or diagnostics, only a few candidate biomarkers have been translated into clinical applications so far. For example, AFP is the most classic biomarker used for HCC detection, but its sensitivity and specificity are unsatisfactory (Galle et al., 2019). In the early 21st century, Li et al. (2022) suggested that hMex-3 might be involved in post-transcriptional regulatory mechanisms, opening up new possibilities for diagnosing HCC. Mex3a is an RNA-binding protein (RBP) that promotes the invasion, proliferation, migration, and viability of cancer cells (Qiu et al., 2022). Its expression has also been shown to be an independent predictor of HCC prognosis (Johnson et al., 2022). On the other hand, recent research has discovered that abnormal DNA methylation is linked to a wide range of human diseases (Nishiyama and Nakanishi, 2021). The DNA methylation status of free cells is similar to that of primary tumor tissue, and early detection can be performed by free cell DNA methylation in liver cancer (Chen et al., 2020). LutaoDu’s article details how gene methylation changes in PBMCs can reflect the severity of colorectal cancer and contribute to accurate early detection of colorectal cancer (Xie et al., 2023). Consequently, there is an urgent need to reveal the underlying connection between Mex3a methylation levels and HCCs, which holds significance for both scientific investigation and clinical diagnosis. In this study, based on a considerable previous effort about the relationship between Mex3a levels and liver cancer development, we propose that a more effective strategy for detecting HCC is the combined test of AFP, Mex3a mRNA levels, and Mex3a promoter methylation levels. It is the first proof that the Mex3a promoter methylation level in PBMCs of HBV-associated HCC patients is decreased compared with that in CHB patients and healthy individuals. The level of Mex3a mRNA in PBMCs of HBV-associated HCC patients is markedly higher than that of CHB patients and normal controls. The PMR value of the Mex3a promoter is negatively correlated with the Mex3a mRNA level. In addition, in HBV-associated HCC, the level of Mex3a promoter methylation in HBV-DNA negative is higher than that in HBV-DNA positive, in >55 years old, in <55 years old, and in those with ascites than in those without. The Mex3a promoter methylation has a significant positive correlation with age, and the Mex3a mRNA level has a clear positive correlation with ALT. The hypomethylation of Mex3a promoter and Mex3a mRNA levels is also an independent risk factor that affects the development of HBV-associated HCC. Meanwhile, based on logistic regression analysis, combined detection of Mex3a promoter methylation level, Mex3a mRNA level, and AFP can improve the diagnostic ability of AFP for HBV-associated HCC. We observed a higher Mex3a promoter methylation level in HCC patients with ascites compared with patients without ascites, which may be because Mex3a can promote tumor progression and affect prognosis (Wang et al., 2023). At the same time, we observed an inconsistency between the absence of significant associations between mRNA levels of Mex3a PMR and factors such as age, HBV-DNA, and ascites, which may be related to post-transcriptional translation (Ying et al., 2023).

The differences in PMR values observed in our study are indeed small, with averages of 0.3% for HCC, 0.9% for CHB, and 2.2% for healthy controls, all falling within a narrow 2% range. Despite the seemingly minor differences, these small variations in promoter methylation are significant for several reasons. First, it is important to note that not all PBMCs undergo reprogramming during cancer or metastasis pathogenesis, which contributes to the “noise” in methylation signals (Andres Houseman et al., 2012). The majority of PBMCs may retain their methylation patterns that are consistent with a non-cancerous state, leading to an overall small change in the average methylation levels (Xu et al., 2017). However, this small signal becomes significant when combined with other biomarkers, such as mRNA levels and AFP. The additive effect of these markers provides a more robust indication of HCC presence, even when individual changes are subtle (Zheng et al., 2018). Second, the small range of methylation differences is biologically meaningful due to the nature of the CpG sites in the promoter region (Jones, 2012). In our study, we tested several CpGs within the Mex3a promoter. While the overall promoter might appear largely unmethylated (e.g., 98% unmethylated in healthy controls), it is the methylation status of a few key CpGs that is critical for gene expression. In healthy controls, these specific CpGs may remain methylated, suppressing Mex3a expression. However, in HCC, even a minor shift, such as the demethylation of these few crucial CpGs, can trigger Mex3a expression. This subtle change, reflected as a 99.7% unmethylated promoter in HCC, is enough to significantly increase Mex3a mRNA levels, contributing to tumorigenesis. This observation underscores the importance of specific CpG sites within the promoter that are essential for gene regulation. While the overall methylation change appears small, the functional impact on gene expression is substantial, making these methylation markers, when combined with mRNA and AFP levels, valuable for distinguishing HCC from other conditions (Shen et al., 2013).

Mex3a expression is an independent predictor of HCC prognosis (Shi et al., 2021). This further demonstrates Mex3a’s potential as a diagnostic marker (Liang et al., 2021). In order to evaluate the diagnostic value of hypermethylation of the Mex3a promoter as a non-invasive biomarker, PBMCs of HCC patients were selected as study specimens, and MethyLight, a high-throughput quantitative methylation assay, was performed with higher sensitivity and specificity than MSP techniques (Yang et al., 2022). The present study demonstrated that ROC curves for Mex3a promoter methylation levels and Mex3a mRNA levels were plotted to evaluate their diagnostic value. The results showed that Mex3a promoter methylation level and Mex3a mRNA level were significantly better than AFP in diagnosis. Combining serum AFP with the PMR value of the Mex3a promoter and Mex3a mRNA level further improves the diagnostic ability of AFP. With 0.824% as the cut-off point, the Mex3a promoter methylation level was 87.8% sensitive and 62.5% specific to distinguish HCC from all populations. Similarly, the Mex3a mRNA level as the cut-off point of 9.005 had a sensitivity of 63.8% and a specificity of 93.6% to distinguish HCC from all patients. If the current recommended clinical cut-off point (20 ng/mL) is used, the sensitivity for AFP is 51.5% and the specificity is 74%. In contrast, the Mex3a promoter PMR combined with Mex3a mRNA levels and AFP had a sensitivity of 75.1% and a specificity of 91.3%. Combined detection of PBMCs’ Mex3a methylation and mRNA levels with serum AFP can significantly improve the diagnostic ability of AFP. Meanwhile, Mex3a promoter methylation levels and mRNA levels were not correlated with AFP. These results suggest that Mex3a promoter methylation levels and mRNA levels may be used as non-invasive diagnostic markers for HCC independent of AFP.

There are several limitations to this experiment. First, the HCC data collection period was brief, and long-term follow-up data prior to and following the incidence were excluded. There is no way to perform a survival study, and no more research has been carried out to determine the predictive usefulness of Mex3a promoter methylation levels in HCC. Second, this study did not explore Mex3a promoter methylation in the context of other pathogens or liver cancer, such as HCV infection, alcohol-related liver disease, or non-alcoholic fatty liver disease. In HCC caused by other factors, Mex3a promoter methylation may exhibit different patterns. Third, the methylation markers tested in the circulating PBMCs were inherently different from those in the original cancer tissue. In this study, we did not detect methylation markers in liver tissue samples, so we could not directly compare the results of liver tissue and PBMCs. Fourth, due to the limited sample size and since just one facility was used to select all the patients, there may have been selection bias. Fifth, the older age of the included HCC patients may have an impact on the wide applicability of Mex3a promoter methylation level as a diagnostic indicator. Finally, there are significant technical challenges with DNA extraction and sodium bisulfite treatment in DNA methylation detection by MethyLight. To support our findings, more multicenter and bigger prospective cohort follow-up studies are required.

## Conclusion

Taken together, we observed that the Mex3a promoter methylation levels, in the context of HBV-associated HCC, are strikingly lower than those of CHB patients and healthy controls, whereas Mex3a mRNA levels in the context of HBV-associated HCC are remarkably higher than in CHB patients and normal individuals. Moreover, it has been established that Mex3a promoter methylation, operating as an independent and potent factor in the development of liver cancer, evinces an inverse association with Mex3a mRNA levels. Notably, as a non-invasive and highly effective biomarker, the methylation level of the Mex3a promoter surpasses AFP in terms of sensitivity and specificity, making it an excellent candidate for the early detection and diagnosis of HCC. Therefore, we firmly believe that Mex3a promoter methylation can serve as a highly reliable and effective method for the detection of HCC, and, as such, we implore healthcare practitioners and researchers alike to prioritize the pursuit of this highly promising avenue of study.

## Data Availability

The original contributions presented in the study are included in the article/Supplementary Material; further inquiries can be directed to the corresponding authors.

## Abbreviations

AASLD, American Association for the Study of Liver Diseases; ALB, aspartate albumin; AST, aminotransferase; ALT, alanine aminotransferase; AFP, alpha-fetoprotein; AUC, the receiver operating characteristic curve; BCLC, Barcelona staging; CHB, chronic hepatitis B; EDTA, ethylene diamine tetraacetic acid; HCC, hepatocellular carcinoma; HBV, hepatitis B virus; HCV, hepatitis C virus; HCs, healthy controls; HBeAg, hepatitis B e surface antigen; NPV, negative predictive value; PBMCs, peripheral blood mononuclear cells; PT-INR, prothrombin time-international normalized ratio; PTA, prothrombin time activity; PPV, positive predictive value; PMR, percentage of methylation reference value; ROC, the receiver operating characteristic; RBP, RNA-binding protein; TBIL, total bilirubin; US, ultrasonography.
